# Variation in the Photoplethysmogram Response to Arousal From Sleep Depending on the Cause of Arousal and the Presence of Desaturation

**DOI:** 10.1109/JTEHM.2024.3349916

**Published:** 2024-01-04

**Authors:** Mieli Luukinen, Henna Pitkänen, Timo Leppänen, Juha Töyräs, Anna Sigridur Islind, Samu Kainulainen, Henri Korkalainen

**Affiliations:** Department of Technical PhysicsUniversity of Eastern Finland 163043 70211 Kuopio Finland; Diagnostic Imaging CenterKuopio University Hospital 60650 70210 Kuopio Finland; School of Electrical Engineering and Computer ScienceThe University of Queensland 1974 Brisbane QLD 4072 Australia; Science Service CenterKuopio University Hospital 60650 70210 Kuopio Finland; Department of Computer ScienceReykjavik University 64401 102 Reykjavik Iceland

**Keywords:** Arousal, delay, obstructive sleep apnea, photoplethysmography

## Abstract

Objective: The aim of this study was to assess how the photoplethysmogram frequency and amplitude responses to arousals from sleep differ between arousals caused by apneas and hypopneas with and without blood oxygen desaturations, and spontaneous arousals. Stronger arousal causes were hypothesized to lead to larger and faster responses. Methods and procedures: Photoplethysmogram signal segments during and around respiratory and spontaneous arousals of 876 suspected obstructive sleep apnea patients were analyzed. Logistic functions were fit to the mean instantaneous frequency and instantaneous amplitude of the signal to detect the responses. Response intensities and timings were compared between arousals of different causes. Results: The majority of the studied arousals induced photoplethysmogram responses. The frequency response was more intense ( ${p} < 0.001$) after respiratory than spontaneous arousals, and after arousals caused by apneas compared to those caused by hypopneas. The amplitude response was stronger ( ${p} < 0.001$) following hypopneas associated with blood oxygen desaturations compared to those that were not. The delays of these responses relative to the electroencephalogram arousal start times were the longest ( ${p} < 0.001$) after arousals caused by apneas and the shortest after spontaneous arousals and arousals caused by hypopneas without blood oxygen desaturations. Conclusion: The presence and type of an airway obstruction and the presence of a blood oxygen desaturation affect the intensity and the timing of photoplethysmogram responses to arousals from sleep. Clinical impact: The photoplethysmogram responses could be used for detecting arousals and assessing their intensity, and the individual variation in the response intensity and timing may hold diagnostically significant information.

## I. Introduction

Obstructive sleep apnea (OSA) is a breathing disorder estimated to affect hundreds of millions of people globally [1]. OSA is defined by the presence of complete and partial obstructions of the upper airways during sleep which are called apneas and hypopneas, respectively [2]. OSA causes cognitive symptoms such as fatigue, daytime sleepiness, and depression as well as issues with concentration and memory, and is associated with an increased risk of cardiovascular diseases [2], [3]. In addition to reducing the quality of life on an individual level [3], [4], on a societal level OSA causes a significant economic burden for example by increasing healthcare costs and accidents and decreasing workplace productivity [3].

According to multiple studies, apneas and hypopneas lead to arousals from sleep in the majority of cases [5], [6], [7], [8]. The arousals cause sleep fragmentation which contributes to the daytime symptoms of OSA [6], [9] and to the development of vascular diseases [10]. The American Academy of Sleep Medicine (AASM) defines an arousal as a sudden shift in the electroencephalogram (EEG) frequency [11]. The magnitude of the EEG activity change during an arousal is affected by the arousal-causing respiratory event severity (aspects such as obstruction type, i.e., apneas vs. hypopneas, and presence of blood oxygen desaturation) [12], [13].

Arousals from sleep elicit sympathetic and cardiovascular responses, including vasoconstriction and heart rate increase [14]. Moreover, arousals contribute to sympathetic overactivity [15], and the nocturnal cardiovascular activity in OSA has been linked to the severity of the OSA symptoms [16]. Furthermore, respiratory arousals induce higher heart rate elevation than spontaneous arousals [17], and the magnitude of the arousal-induced heart rate elevation is correlated with the intensity of the EEG changes in arousals [18]. The peak of the heart rate elevation [19], and the vasoconstriction [20] occur at a few seconds delay relative to the beginning of the arousal or the end of the arousal-causing respiratory event. No significant differences in the cardiovascular responses have been found either between arousals caused by apneas and hypopneas [20], or between auditorily induced arousals in controlled normoxia and hypoxia [21]. However, in these studies the number of subjects was rather low, respectively 12 and 11 subjects. Even though the timing of the cardiovascular response has been studied relative to the respiratory event [19], the factors that affect the timing relative to the cortical arousal have to our knowledge not been extensively studied.

The first aim of this study is to evaluate the effect of the respiratory event type on the respiratory arousal-related cardiovascular responses, in a larger cohort ( $n = 876$ subjects) than in the previous studies. The second aim is to compare the post-arousal delays of these cardiovascular responses between different respiratory event types.

In this study, it is hypothesized that arousal-causing apneas, compared to hypopneas, and respiratory arousals compared to spontaneous arousals induce stronger photoplethysmogram (PPG) frequency and amplitude responses to the arousal. Another hypothesis is that similarly the presence of desaturation during the arousal-causing respiratory event induces stronger arousal responses. Furthermore, it is hypothesized that the delays of these responses relative to the EEG arousal are decreased by the same aforementioned factors. Preliminary results of this work have been previously reported in a conference abstract [22].

## II. Methods

### Dataset

In this study, a clinical dataset consisting of polysomnography (PSG) data of 933 subjects with suspected OSA was analyzed retrospectively. The data was collected between 2015 and 2017 at the Princess Alexandra Hospital in Brisbane, Australia. The data collection and its utilization in research was approved by the Metro South Human Research Ethics Committee (HREC/16/QPAH/021 (January 9^th^, 2016) and LNR/2019/QMS/54313 (June 10^th^, 2019)). The PSGs were recorded using the Compumedics Grael acquisition system (Compumedics, Abbotsford, Australia). The arousals, sleep stages, desaturations, and respiratory events were scored by experienced sleep technicians, who are regularly subjected to an inter-rater agreement protocol, using Compumedics Profusion 4.0 software and following the 2012 scoring rules of AASM [11]. Respiratory events, arousals, and sleep stages were scored manually, whereas blood oxygen desaturations were scored utilizing automated tools. The PSGs were pseudonymized to protect the subjects’ privacy.

**FIGURE 1.**
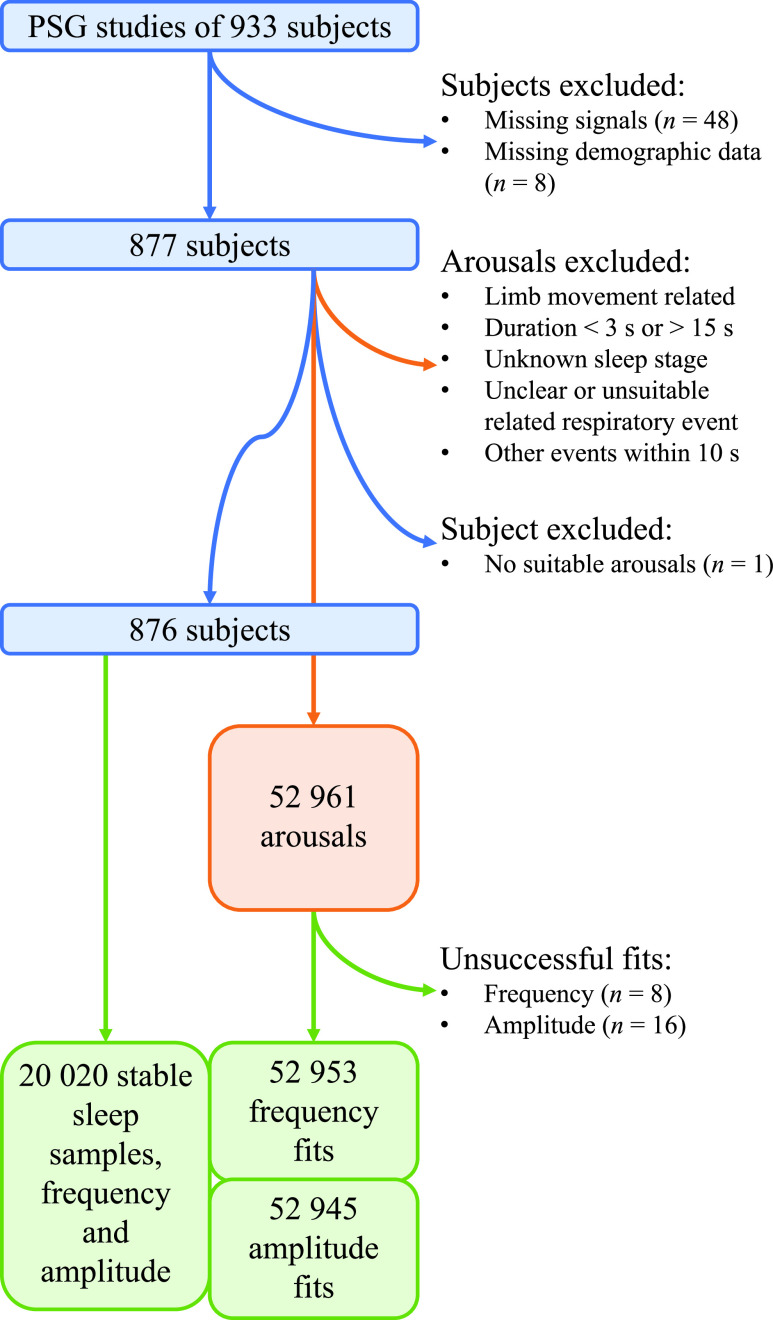
The research data workflow. PSG, polysomnography.

Subjects were excluded from the analyses if any of the required sleep data (PPG signal or scorings of arousals, respiratory events, or sleep stages) were missing ( $n$ = 48) or if the subject’s demographic information was incomplete ( $n$ = 8). Arousals were excluded from the study if their duration was below 3 s or over 15 s, if neither the epoch where the arousal occurred nor the previous one had a scored sleep stage, or if another arousal or a respiratory event other than the assumed cause of the arousal was observed within 10 s of the start or the end of the arousal. In the case of respiratory arousals, those that could not be uniquely associated with an obstructive respiratory event, such that the event ended no earlier than five seconds before the arousal started and at the latest during the arousal, or if the associated event lasted less than 10 s, were further excluded. Additionally, only obstructive respiratory and spontaneous arousals were investigated, and other arousals were excluded. If all arousals from a subject were excluded, the subject was removed from the subsequent analyses ( $n$ = 1). After the exclusion, a total of 23 007 respiratory arousals and 29 954 spontaneous arousals from 876 subjects were included in the subsequent analyses. The data exclusion workflow is shown in Fig. 1. After the exclusion, the included arousals were grouped based on whether the arousal was spontaneous or respiratory-related. Moreover, the respiratory arousals were further classified based on the type of the arousal-causing respiratory event and the presence or absence of a ≥3% blood oxygen desaturation event. The included dataset and the numbers of arousals of each arousal type are described in Table 1. In addition, the arousal types are described in more detail in Table S.I in the supplementary material.

**TABLE 1:** Demographical Information and Sleep Statistics of the Subjects, and Arousal Types

	$n$	% of the population
Male	481	54.9
Female	395	45.1
	Median	Interquartile range
Age (years)	55.9	44.7–65.7
BMI (kg/m^2^)	34.4	29.4–40.4
TST (h)	4.2	5.1–6.0
WASO (h)	1.7	1.0–2.5
AHI (1/h)	18.6	8.3–39.2
ArI (1/h)	26.3	17.9–41.3
ArRI (1/h)	11.1	4.5–24.4
Stage R (%)	17.1	11.9–22.1
NREM (%)	82.9	77.9–88.1
Stage N1 (%)	11.0	6.8–18.8
Stage N2 (%)	48.4	41.4–56.1
Stage N3 (%)	18.4	9.6–27.0
Type of arousal	$n$	% of studied arousals
Apnea, Desaturation	2287	4.3
Apnea, no Desaturation	935	1.8
Hypopnea, Desaturation	8214	15.5
Hypopnea, no Desaturation	11571	21.8
Spontaneous	29954	56.6

BMI, body mass index; TST, total sleep time; WASO, wake after sleep onset; AHI, apnea-hypopnea index; ArI, arousal index; ArRI, respiratory arousal index; Stage R, rapid eye movement (REM) sleep; NREM, non-REM sleep; Stage N1, NREM 1 sleep; Stage N2, NREM 2 sleep; Stage N3, NREM 3 sleep; Desaturation, ≥3% blood oxygen desaturation. Sleep stage percentages were calculated from TST. For other than spontaneous arousals, the arousal type is the type of the arousal-inducing respiratory event.

A more detailed description of the data exclusion is presented in the supplementary material.

### Signal Processing

The data was exported from the Profusion software and processed and analyzed with Python 3.8.5 (Python Software Foundation, DE, USA), using the SciPy [23] and NumPy [24] packages. The nocturnal PPG of each subject was decimated to a sampling rate of 64 Hz. For each EEG arousal, a segment of the PPG signal was extracted, from 10 s before the arousal start to 10 s after the end of the arousal.

For each segment, a spectrogram was computed with a two-second boxcar window and with a zero-padded fast Fourier transform with a length of 16 times the sampling rate for a smoothened frequency domain. Each window had 128 samples, with an overlap of 127 samples between consecutive windows to preserve the sampling rate. The mean instantaneous frequency ( $\bar {f}_{\text {inst}}$) was then defined for each segment as the weighted average of the frequency, using the spectrogram power as the weight. This can be expressed as follows:

\begin{align*} \bar {f}_{\text {inst}}(t) = \frac {\sum _{f \in F} f \cdot \text {PSD}_{f}\left({PPG\left({\left[{t-1 \text {s}, \ldots, t+\frac {63}{64} \text {s}}\right]}\right)}\right)}{\sum _{f \in F}\text {PSD}_{f}\left({PPG\left({\left[{t-1 \text {s}, \ldots, t+\frac {63}{64} \text {s}}\right]}\right)}\right)}, \tag{1}\end{align*}

where $t$ is time in seconds, with a step size of $\frac {1}{64}$ s, $F$ is the range from 0 Hz to 32 Hz with a step size of $\frac {1}{16}$ Hz, $\text {PSD}_{f}(\vec {x})$ is the power spectral density of signal $\vec {x}$ in the frequency bin centered on $f$ and $PPG$ is the photoplethysmogram signal. The instantaneous amplitude was derived for each segment by subtracting the minimum PPG value within a two-second moving window from the maximum, similarly overlapping the windows to preserve the sampling rate. The frequency and amplitude characteristics were then smoothed with a moving average filter with a two-second window. After the derivation of the characteristics and the filtering, each segment had a length of the arousal duration plus 16 s.

For the comparison of the frequency and amplitude levels before and after arousals to those during stable sleep, samples of stable sleep were also analyzed. The set of samples was formed such that it obtained a representation of each subject and had as similar as possible distribution of sleep stages to the set of respiratory arousals studied. For each subject, a maximum amount of 10 s periods without any scored events or transitions between sleep stages were separated. Those periods were grouped by the sleep stage, and each group was randomly permutated, and as many samples were collected as that subject had respiratory arousals in the sleep stage in question that were included in the analyzes. In case the amount of stable sleep periods was smaller than the corresponding number of respiratory arousals of the subject, all stable sleep samples were included from that subject. A total of 20 020 stable sleep samples were collected. For each sample, the mean instantaneous frequency and instantaneous amplitude were derived similarly to the arousal samples, and a mean over the duration of the sample was taken of both characteristics.

A more detailed description of the signal processing is presented in the supplementary material.

### Arousal Response Detection

To detect the signal responses related to the arousals while separating them from artefacts, a function consisting of three consecutive logistic functions with alternating signs was fitted to the derived frequency and amplitude characteristics. The function, a compromise between limited model complexity and power for detecting changes, was of the form

\begin{align*} f(t)=a+\frac {b_{1}}{1+e^{-5(t-c_{1})}}-\frac {b_{1} b_{2,\text {rel}}}{1+e^{-5(t-c_{2})}}+\frac {b_{1} b_{3,\text {rel}}}{1+e^{-5(t-c_{3})}}, \tag{2}\end{align*}

where

**FIGURE 2.**
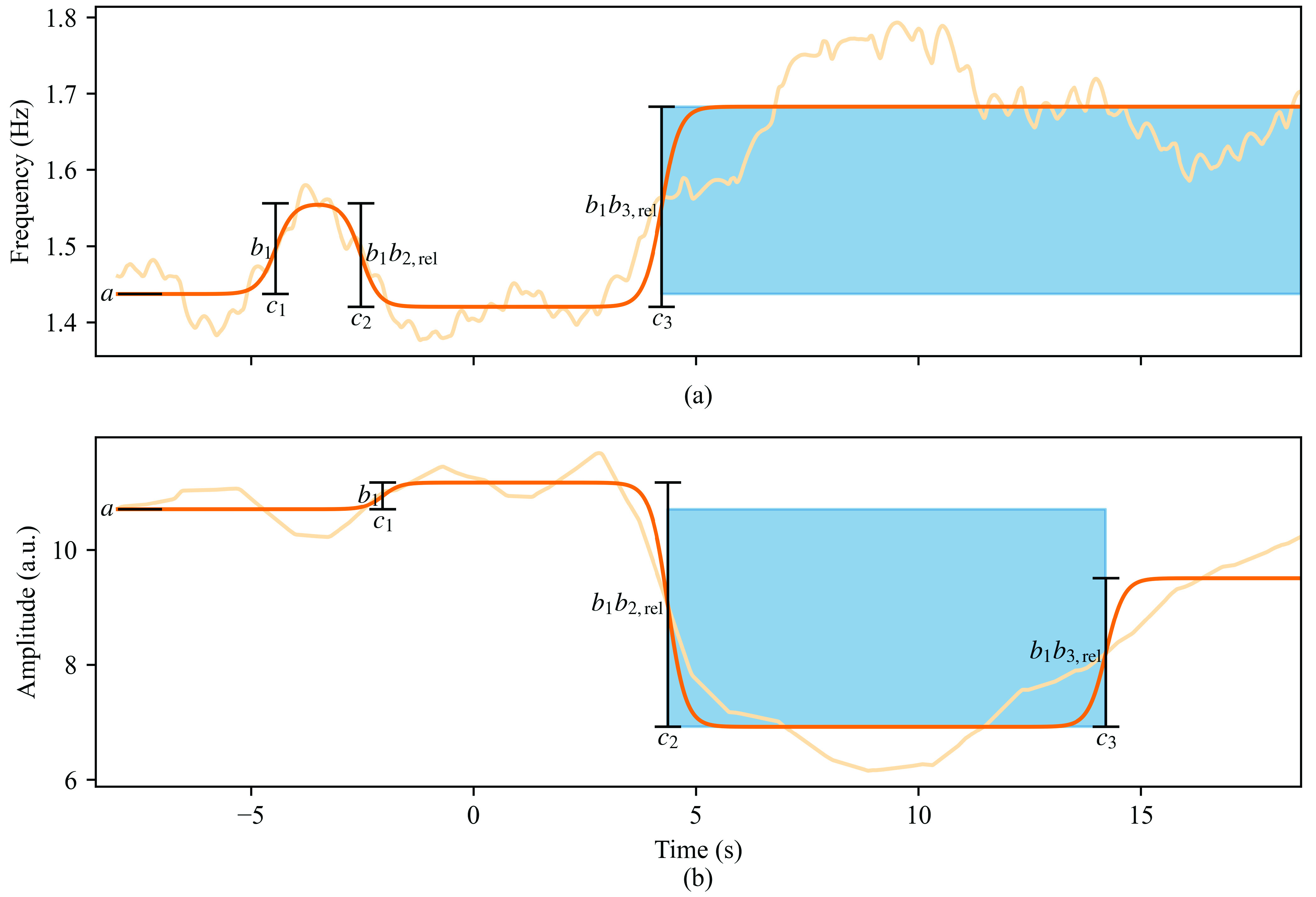
Example of fitting (2) to the photoplethysmogram signal characteristics ((a) mean instantaneous frequency (b) instantaneous amplitude) before, during and after an arousal and detecting the arousal responses. The light orange line shows the smoothed signal characteristic, the darker orange line is the fitted function and the blue rectangle marks the detected response. $a$ is the fitted value in the beginning of the time window, $b_{1}$, $b_{1}b_{2,\text {rel}}$, and $b_{1}b_{3,\text {rel}}$ are the magnitudes of the fitted logistic functions and $c_{1}$, $c_{2}$, and $c_{3}$ are their times. The time in the figure is relative to the EEG arousal start. Both (a) and (b) depict data from the same arousal. (a) $a = 1.44$ Hz, $b_{1} = 0.120$ Hz, $b_{1}b_{2,\text {rel}} = 0.136$ Hz ( $b_{2,\text {rel}} = 1.14$), $b_{1}b_{3,\text {rel}} = 0.263$ Hz ( $b_{3,\text {rel}} = 2.20$), $c_{1} = -4.45$ s, $c_{2} = -2.53$ s ( $c_{2,\text {rel}} = 0.0459$), $c_{3}= 4.23$ s ( $c_{3,\text {rel}} = 0.301$). The detected response starts at $c_{3}$, has magnitude of 0.247 Hz and no recovery from it is observed. (b) $a = 10.7$ a.u., $b_{1} = 0.470$ a.u., $b_{1}b_{2,\text {rel}} = 4.25$ a.u. ( $b_{2,\text {rel}} = 9.02$), $b_{1}b_{3,\text {rel}} = 2.58$ a.u. ( $b_{3,\text {rel}} = 5.49$), $c_{1} = -2.05$ s, $c_{2} = 4.37$ s ( $c_{2,\text {rel}} = 0.307$), $c_{3} = 14.2$ s ( $c_{3,\text {rel}} = 0.723$). The detected response starts at $c_{2}$, has magnitude of 3.77 a.u. and recovery from it is observed at $c_{3}$.

\begin{align*} c_{2}&=c_{1}+1+c_{2,\text {rel}} (t_{\text {max}}-c_{1}-3), \\ c_{3}&=c_{2}+1+c_{3,\text {rel}} (t_{\text {max}}-c_{2}-2),\end{align*}

and $t$ is the timepoint relative to the EEG arousal start, $t_{\text {max}}$ is the endpoint of the time window, $a$ is the fitted value at the beginning of the window, $b_{1}$ is the magnitude of the first logistic function, and $b_{2,\text {rel}}$ and $b_{3,\text {rel}}$ are the magnitudes of the second and third logistic functions, respectively, proportional to $b_{1}$. Furthermore, $c_{1}$, $c_{2}$, and $c_{3}$ are the timepoints of the logistic functions, $c_{2,\text {rel}}$ is the timepoint of the second logistic function proportional to the range between one second after the first logistic function and two seconds before the time window end, and $c_{3,\text {rel}}$ is the timepoint of the third logistic function proportional to the range between one second after the second logistic function and one second before the time window end. The constant logistic growth rate (value 5) was chosen based on visual examination of sample arousal responses, so that it would be close in scale to the typical changes in the characteristics. The parameters to fit were $a$, $b_{1}$, $b_{2,\text {rel}}$, $b_{3,\text {rel}}$, $c_{1}$, $c_{2,\text {rel}}$, and $c_{3,\text {rel}}$. Examples of the fit are shown in Fig 2.

The least squares method was used for fitting the function. Table 2 shows the fit ranges and the initial guesses for the parameters. The initial guess for $a$ is the median value before arousal start, as it is the value in the fit beginning. The variable $b_{1}$ is initialized as 0, so it is biased neither towards positive nor negative first change. The second and third steps may have been smaller or larger than the first one, so $b_{2,\text {rel}}$ and $b_{3,\text {rel}}$ were initialized as 1. As the initial guess for the fitting parameters may affect the fit results, there were multiple initial guesses for $c_{1}$, $c_{2,\text {rel}}$, and $c_{3,\text {rel}}$ around the parameter ranges to ensure a proper fit is found instead of just a local optimum. The guesses for $c_{1}$ were centered around the EEG arousal start, and those for $c_{2,\text {rel}}$ around the halfway point after the first change. The guesses for $c_{3,\text {rel}}$ were larger values to detect very late changes. The fitting was repeated with all the different combinations of initial guesses, and the fit minimizing the sum of the squared residuals was chosen. Arousals that were not successfully fitted were further discarded from the dataset (Fig. 1). To determine the PPG response delays relative to the EEG arousal start, the frequency and amplitude levels before and after the response, and whether the characteristics recovered from the response within the studied time window, each fit of (2) was categorized to one of the 16 classes detailed in Table S.II and visualized in Fig. S.1 in the supplementary material. This categorization was based on the considerability of the fitted steps as follows. To limit the effects of noise, each step was deemed considerable if its magnitude was higher than 5% of the mean fitted value. The magnitudes of the frequency increases and amplitude decreases in response to arousals were calculated as the difference between the levels before and after the responses. Examples of the detected responses are shown in Fig 2.

**TABLE 2:** The Ranges and Initial Guesses of the Fit Parameters Utilized in (2)

Parameter	Range	Initial guesses
$a$	$[0, y_{\text {max}}]$	median value before arousal start
$b_{1}$	$[-y_{max}, y_{max}]$	0
$b_{2,rel}$	$[0, \infty]$	1
$b_{3,rel}$	$[0, \infty]$	1
$c_{1}$	$[-t_{min}+1, t_{max}-3]$	−5, 0, 5
$c_{2,rel}$	$[{0, 1}]$	0.3, 0.5, 0.7
$c_{3,rel}$	[0, 1]	0.5, 0.7, 0.9

$y_{max}$, the largest value of frequency or amplitude within the time window; $t_{min}$, $t_{max}$, the beginning and the end times of the time window, respectively, in seconds relative to the EEG arousal start.

A more detailed description of the response detection is presented in the supplementary material.

### Statistical Analysis

For most of the analyses, the arousals were grouped by the arousal type, separating respiratory arousals from spontaneous arousals. The respiratory arousals were further divided into four groups; arousals caused by obstructive apneas were separated from those caused by hypopneas, and in both cases additionally grouped by whether the arousal-causing respiratory event was accompanied by a blood oxygen desaturation of at least three percentage points. Due to each group containing arousals from the same subjects and thus the samples not being statistically independent, Wilcoxon signed-rank test was used for the statistical inference analyses, iteratively as follows. In each test between two arousal groups, for each subject, the number of arousals equal to that in the group with fewer of them was randomly sampled from the other group. This sample was then randomly paired with arousals from the same subject in the other group. The samples from each subject were then combined for the test. The Wilcoxon signed-rank test was then repeated 1 000 times, with the random sampling done again for each iteration. The median $p$-value of the iterations was then used as the measure of statistical significance. Due to the large number of comparisons in this study, $p = 0.01$ was used as the significance threshold.

For each arousal group, the proportions of arousals with and without an observed PPG response were calculated for both response types. Additionally, the proportions of arousals with and without an observed recovery from the arousal response were calculated among the arousals with responses. The arousal types were compared by calculating the relative risk of not detecting a response or a recovery, between each pair of arousal types. For the relative risks, 99% confidence intervals were calculated and compared with unity. Furthermore, to assess the effect of the response magnitude on the likelihood of an observed recovery, the arousals with an observed response were grouped by the presence of recovery, and the response magnitudes between the groups were compared using the Wilcoxon signed-rank test.

The response magnitudes were compared between different arousal types, i.e., arousals caused by different kinds of respiratory events using the Wilcoxon signed-rank test. The frequency and amplitude values before and after the responses were compared with the Wilcoxon signed-rank test to the baseline, defined as the median frequency and amplitude of the stable sleep samples. The comparison of the response delays relative to the EEG arousal start was also done with the Wilcoxon signed-rank test. The relationships between response magnitudes and delays were assessed using the Pearson correlation.

## III. Results

### Arousal-Induced Responses and Recoveries Observed by the Model

An increase in the PPG signal’s mean instantaneous frequency was observed in 74.5% and a decrease in the instantaneous amplitude in 82.0% of the studied arousals. Both of these responses where observed in 66.2% of the studied arousals, and only 9.7% of the arousals were not associated with either of the responses. A recovery from the responses towards the pre-arousal level was detected after 66.4% of the frequency increases and 50.0% of the amplitude decreases.

**FIGURE 3.**
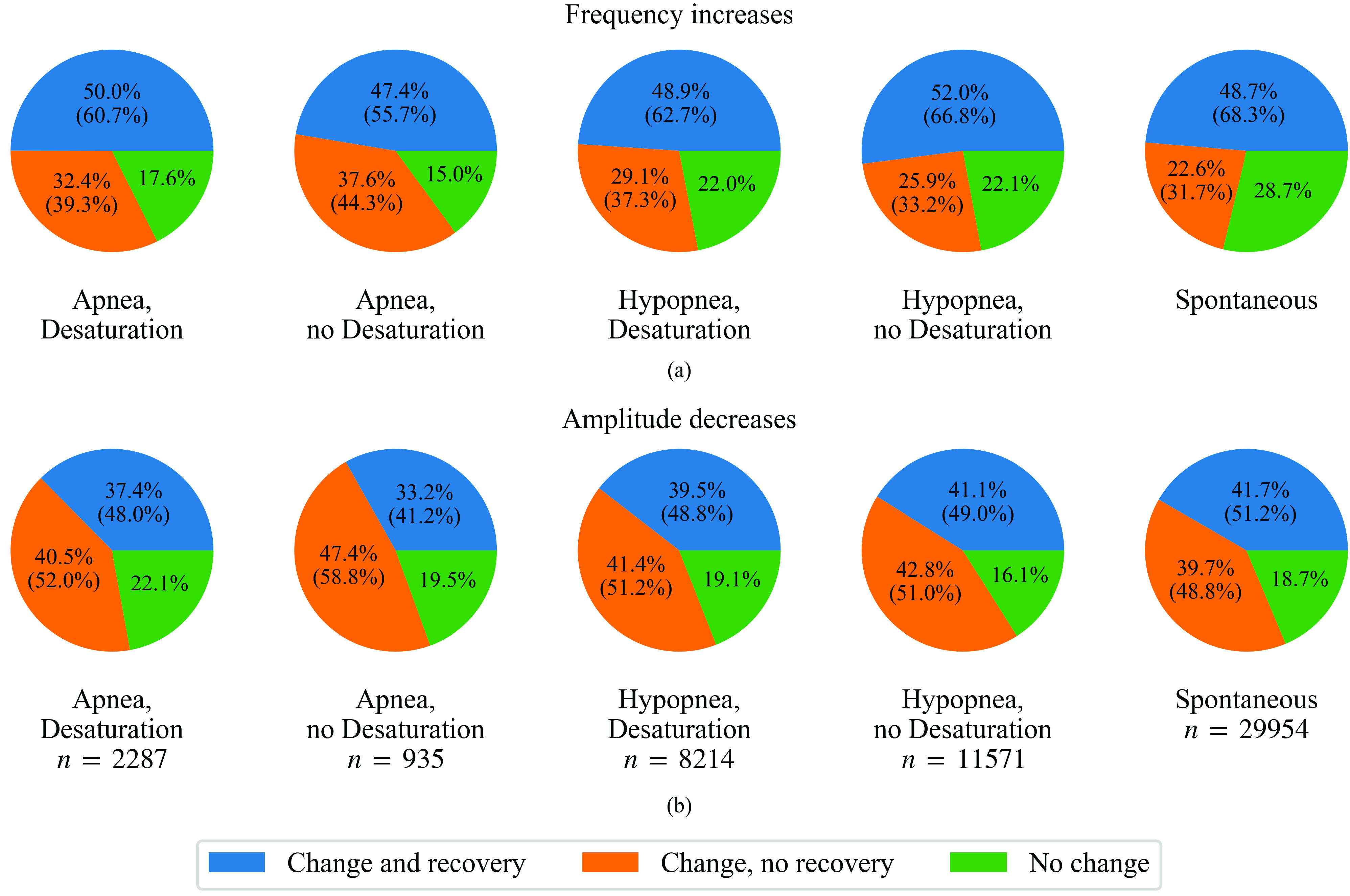
Proportions of arousals with or without related photoplethysmography signal (a) frequency increase or (b) amplitude decrease, as well as with or without a recovery from these changes grouped based on the cause of arousal. The percentages in parentheses are the portions relative to the sum of those arousals where change was observed. For other than spontaneous arousals, the arousal type is the type of the arousal-inducing respiratory event. Desaturation, ≥3% blood oxygen desaturation.

**FIGURE 4.**
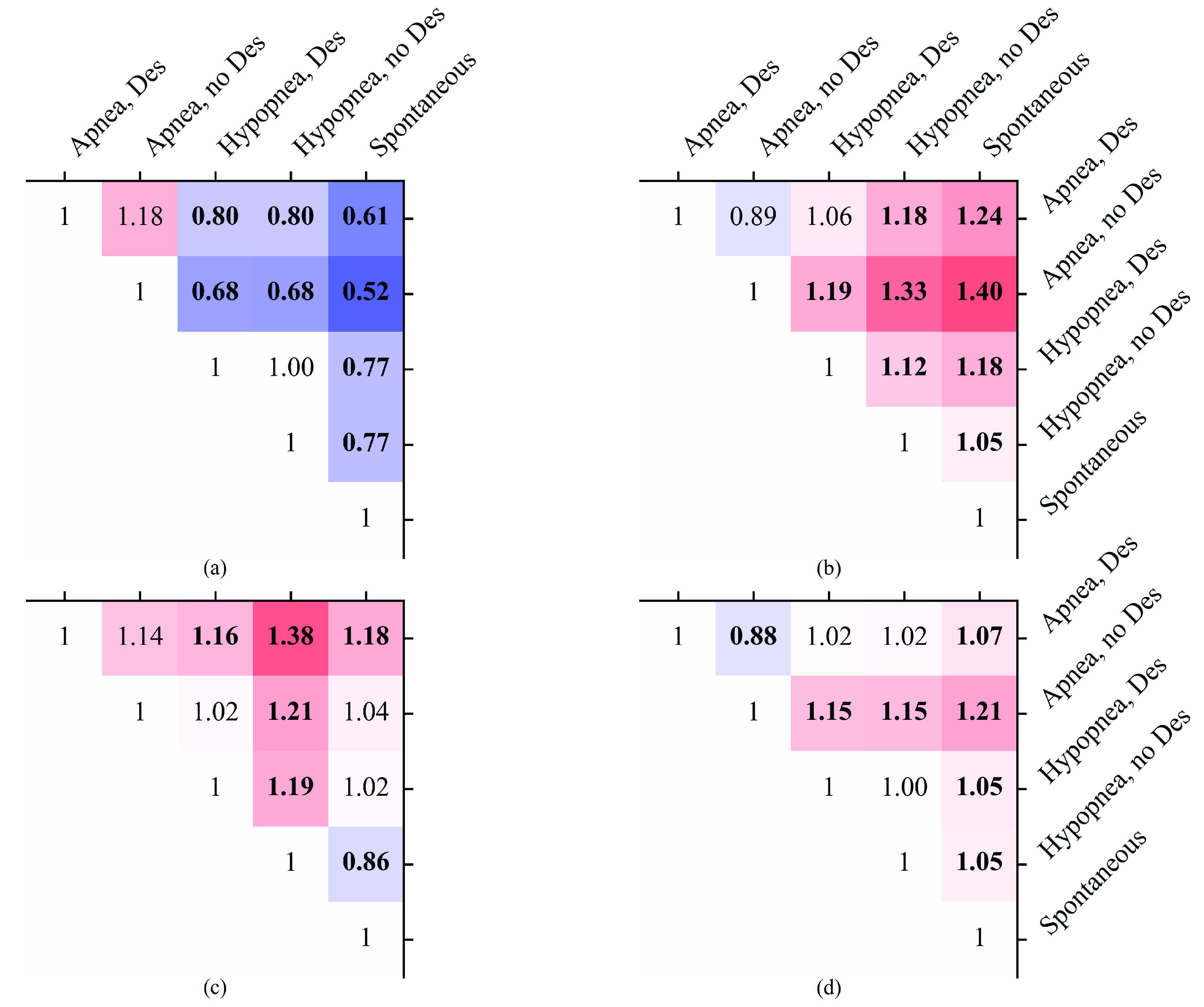
Relative risks of not detecting (a) frequency increase (b) frequency recovery (c) amplitude decrease (d) amplitude recovery between different arousal types. Blue tint, and correspondingly value below 1 indicates lower risk for the arousal type of the row relative to that of the column, and red tint and value above 1 is the opposite. The value is marked in bold if the 99% confidence interval of the relative risk does not contain value 1. For other than spontaneous arousals, the arousal type is the type of the arousal-inducing respiratory event. Des, ≥3% blood oxygen desaturation.

Out of all studied arousals, frequency increases were most likely to be observed following arousals caused by apneas (83.1%, relative risks of not observing an increase compared to non-apnea arousal types are all below unity with >99% confidence), and least likely following spontaneous arousals (71.3%, >99% confidence) (Figs. 3 and 4). Frequency increases were observed to recover towards baseline most likely after spontaneous arousals (68.3%, >99% confidence) and least likely after apnea-related arousals without desaturations (55.7%, >99% confidence compared to arousal types other than apnea with desaturation).

Amplitude decreases were more likely to be observed related to hypopnea- than apnea-induced arousals (82.7% vs. 78.6%, >99% confidence when comparing with matched desaturation presence). Among hypopneas the observation was more likely when a desaturation was not present than in the presence of desaturation (83.9% vs 80.9%, >99% confidence). Similar difference was seen among apneas (80.5% vs. 77.9%), but there the 99% confidence was not reached. Recovery from the amplitude decrease was more likely to be observed related to spontaneous than respiratory arousals (51.2% vs. 48.5%, >99% confidence), and least likely related to arousals caused by apneas without desaturation (41.2%, >99% confidence).

The median amplitude response magnitudes were greater ( $p < 0.001$) when no recovery was observed compared to when the amplitudes recovered (2.0 a.u. vs. 1.7 a.u.). For the median frequency response, the difference between the groups without and with a recovery (0.24 Hz vs. 0.23 Hz) implied a similar relationship, but the Wilcoxon signed-rank test gave a significant ( $p < 0.001$) difference in the opposite direction, indicating a greater change when a recovery was observed.

### Frequency and Amplitude Response Magnitudes, and Levels Before and After the Arousal-Induced Responses

**FIGURE 5.**
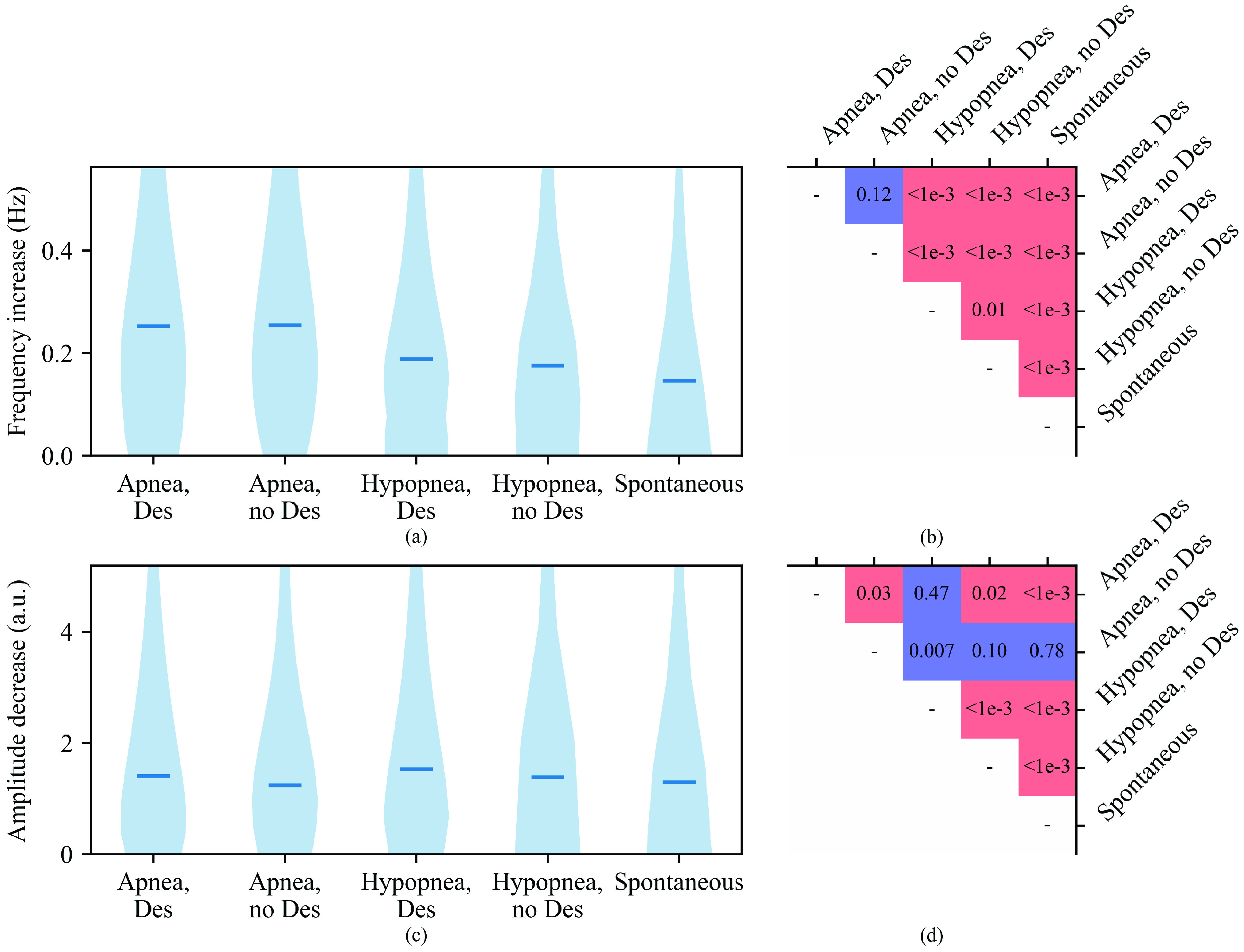
Magnitude distributions of (a) frequency increases and (c) amplitude decreases for different arousal types, with medians marked as horizontal lines, and significance levels for their differences in (b) and (d), respectively. The vertical axes of (a) and (c) have been limited to show data from 0 to the 90th percentile. Red squares indicate that the absolute median is larger for the arousal type of the row compared to that of the column, and blue is the opposite. The $p$-values were calculated with the iterated one-sided Wilcoxon signed-rank test. For other than spontaneous arousals, the arousal type is the type of the arousal-inducing respiratory event. Des, ≥3% blood oxygen desaturation.

Arousal-induced PPG frequency increases were smaller ( $p < 0.001$) in spontaneous arousals (median 0.15 Hz) compared to respiratory arousals (0.19 Hz), and larger ( $p < 0.001$) following arousals caused by apneas than by hypopneas (0.25 Hz vs. 0.18 Hz) (Figs. 5a and 5b). When compared with the stable sleep baseline (1.59 Hz) the frequency levels prior to the arousal-induced increases were significantly below the baseline in the case of respiratory arousals ( $p = 0.004$ for apneas without desaturation and $p < 0.001$ for other cases) (Table 3). The frequency levels before the arousal-induced increases were not significantly different from the baseline in the case of spontaneous arousals ( $p = 0.018$). The post-increase frequency levels were significantly ( $p < 0.001$) higher than the baseline for all types of arousal, with the highest values related to arousals caused by apneas.

**TABLE 3:** Relative Median Frequency and Amplitude Levels Before and After Arousal-Induced Photoplethysmogram Responses

Type of arousal	$n$	Frequency		Amplitude	
		Before	After	Before	After
Apnea, Des	2477	−2.23%	+16.5%	+15.8%	−14.2%
Apnea, no Des	1009	−1.20%	+16.7%	−7.82%	−31.2%
Hypopnea, Des	8305	−0.26%	+14.3%	+22.0%	−9.78%
Hypopnea, no Des	11613	−2.63%	+11.1%	+3.02%	−25.8%
Spontaneous	27359	+1.48%	+12.7%	+8.20%	−17.9%

The frequency and amplitude levels are shown as relative differences from stable sleep median. The significance of the differences was assessed with the Wilcoxon signed-rank test, and differences with $p < 0.01$ have been marked in bold. The levels before and after responses were defined based on the fitting of (2) as detailed in the supplementary Table S.I For other than spontaneous arousals, the arousal type is the type of the arousal-inducing respiratory event. Des, ≥3% blood oxygen desaturation.

Respiratory arousal-induced PPG amplitude decrease was greater ( $p < 0.001$) when the arousal-causing respiratory event was accompanied with desaturation (median 1.50 a.u.), compared to arousals of other types (1.37 a.u. for respiratory arousals without desaturation and 1.29 a.u. for spontaneous arousals) (Figs. 5c and 5d). However, when compared separately among apneas, the difference between those with and without desaturation was not statistically significant ( $p = 0.03$). Moreover, when compared with stable sleep baseline (6.32 a.u.), the amplitude levels before the arousal-induced decreases were significantly ( $p < 0.001$) increased relative to the baseline in all the other cases except apneas without blood oxygen desaturation (Table 3). Furthermore, the post-decrease amplitude levels were significantly ( $p < 0.001$) below the baseline for arousals of all types, with lowest levels associated with the arousals caused by respiratory events without desaturation.

### Delay Between EEG Arousal and PPG Response

**FIGURE 6.**
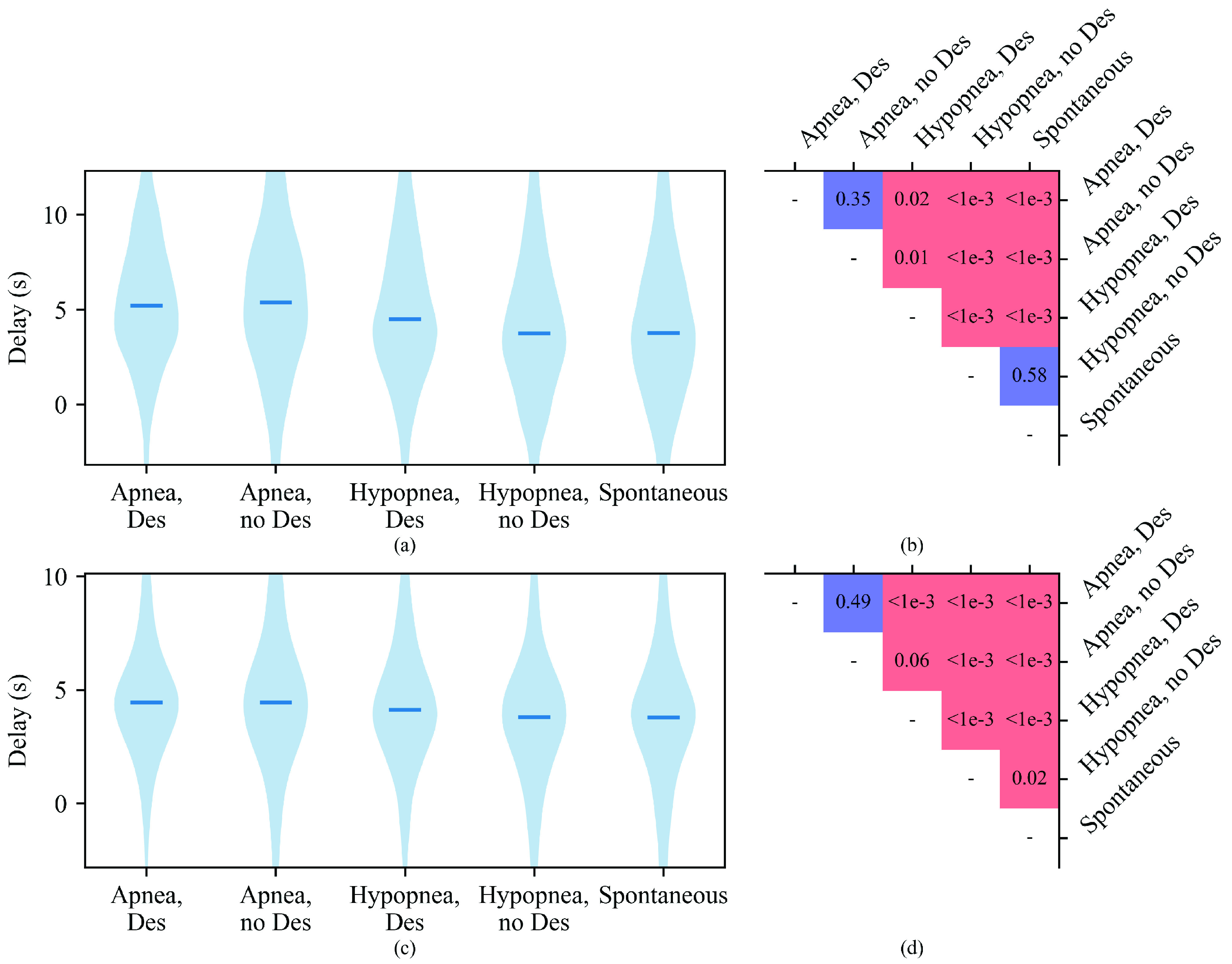
Delay distributions of (a) frequency increases and (c) amplitude decreases in photoplethysmogram signal from the electroencephalogram arousal start for different arousal types, with medians marked with horizontal lines, and significance levels for their differences in (b) and (d), respectively. The vertical axes of (a) and (c) have been limited between 5th and 95th percentiles of the whole data. Red squares indicate that the absolute median is larger for the arousal type of the row compared to that of the column, and blue is the opposite. The $p$-values were calculated with the iterated one-sided Wilcoxon signed-rank test. For other than spontaneous arousals, the arousal type is the type of the arousal-inducing respiratory event. Des, ≥3% blood oxygen desaturation.

The longest delays of the frequency responses were observed after arousals caused by apneas in general or hypopneas with desaturation (median 4.7 s), with shorter delays observed after arousals caused by hypopneas without desaturation and spontaneous arousals (3.8 s, with $p < 0.001$ for each pair of causes with long and short delays) (Fig. 6). The amplitude responses had longest delays observed after arousals caused by apneas (median 4.4 s), followed by arousals caused by hypopneas ( $p < 0.001$ when comparing apneas with desaturation to both types of hypopneas and when comparing apneas and hypopneas without desaturation, but no significant difference between apneas without desaturation and hypopneas with desaturation). In the case of hypopneas, the amplitude response delays were further prolonged ( $p < 0.001$) in the presence of blood oxygen desaturations (4.1 s vs. 3.8 s). Spontaneous arousal-induced amplitude responses displayed shorter delays than those caused by respiratory arousals (median 3.8 s), with no significant difference to the delays after arousals caused by hypopneas without desaturations but $p < 0.001$ when comparing to other causes. Weak but statistically significant ( $p < 0.001$) correlations were found between the delay and the magnitude of the frequency increase (correlation coefficient $\rho = 0.18$), and between the delay and the amplitude decrease ( $\rho = -0.019$).

## IV. Discussion

### Interpretation of the Results

In this study, the majority of the studied arousals were found to elicit responses in the mean instantaneous frequency and the instantaneous amplitude of the PPG signal. The frequency response was found to be stronger for respiratory than spontaneous arousals and stronger for arousals caused by apneas than those caused by hypopneas. The amplitude response was found to be stronger in the presence of a blood oxygen desaturation. The delays of these responses relative to the EEG arousals were found to be the longest for arousals caused by apneas and the shortest for spontaneous arousals and arousals caused by hypopneas without a desaturation.

The changes in PPG characteristics associated with cardiovascular responses were observed following the majority of the arousals studied. Due to the considerability limits for the step sizes used in the categorization of the fits of (2), it is possible that when responses were not detected they simply were weaker. The frequency response was least likely to be observed for spontaneous arousals and less likely for arousals induced by hypopneas than apneas. As the frequency responses to respiratory arousals were greater than to spontaneous arousals, and greater following apneas than hypopneas, this supports the idea that the responses are less likely to be observed in the cases where they are generally weaker. For the amplitude response there is evidence of the opposite, as respiratory events with desaturations are associated with stronger responses but are also more likely to show no response.

Approximately two thirds of the frequency increases and half of the amplitude decreases detected were observed to recover towards the pre-response levels. The portions not observed to recover may be explained by the cardiovascular system recovering from the arousal response so gradually that most of these recoveries occurred outside the studied time window. Arousals caused by apneas were the least likely to show a recovery from the frequency response, whereas spontaneous arousals were associated with recoveries the most. As the former were also associated with stronger frequency responses, this can imply that larger frequency responses may either be less likely to recover at all during the sleep, or they may recover more slowly, i.e., outside the chosen time window. However, the more direct analysis of this relationship gave unclear results, with the statistical test implying the opposite relationship to that implied by the median difference. Conversely, for the amplitude response, the direct comparison between the groups with and without an amplitude recovery supports the idea that greater responses are less likely found to recover within the time window. While the recovery mechanisms may be different for the frequency and amplitude responses, in general it seems that greater responses are more likely to persist longer time.

As the frequency of the PPG signal is known to correspond to heart rate [25], the present finding of increased mean instantaneous PPG frequency after arousals is in concordance with the elevated heart rate response to an arousal stated in the literature [17]. Moreover, the present finding of a higher PPG frequency after respiratory than spontaneous arousals is also consistent with the heart rate responses to these arousals [17]. The frequency increase was also found to be significantly larger after arousals caused by apneas compared to those caused by hypopneas. This supports our hypothesis, but disagrees with the results of Haba-Rubio et al. [20], who found no significant difference between cardiovascular responses to arousals related to apneas and hypopneas. However, the present study has over 70 times more subjects than the previous study [20], increasing the statistical credibility of the present results. In respiratory arousals, heart rate decrease relative to the stable sleep baseline was found to precede the increase, which is consistent with bradycardia found to be present during the obstructive respiratory events [20].

It is assumed that an amplitude decrease in the PPG signal reflects vasoconstriction [26]. Supporting our hypothesis, the decreasing PPG amplitude response to arousals was stronger in the presence of desaturation in the case of hypopneas. A similar relationship was observed in the case of apneas but turned out not to be statistically significant. This effect is contrary to the findings of Catcheside et al. [21], who found no significant difference in the cardiovascular responses to arousals between normoxia and hypoxia. However, their study setting was very different, with controlled constant oxygen levels and auditorily induced arousals in only 11 subjects. It is noteworthy, that despite the larger amplitude decreases in the presence of a desaturation, the post-response amplitude levels were lower when there was no desaturation present. This can be due to the already lower amplitude levels even before these arousal responses (Table 3).

Response delays behaved similarly for both the frequency and amplitude responses. In general, the longest delays were associated with arousals caused by apneas and the shortest ones with spontaneous arousals and arousals caused by hypopneas without a desaturation. Thus, contrary to the hypothesis, stronger respiratory events (apneas compared to hypopneas and among hypopneas those with desaturation compared to those without) prolonged the PPG response to the related arousal, rather than shortened it. As arousals related to stronger respiratory events were also noticed to induce stronger responses, these responses may take longer for the cardiovascular system to actualize. However, the correlations between the response magnitudes and delays were low, which does not support this idea. Additionally, the magnitude-delay-dependence of the responses is similar for both frequency increases and amplitude decreases, instead of following the specific effects of arousal type on the magnitudes of the responses. Nevertheless, a non-linear connection may still exist between the response magnitude and delay, which may have been concealed for example by individual variation in the delays. On the other hand, Azarbarzin et al. [19] found the location of the heart rate peak after the respiratory event to be unaffected by the severity of the event. This may be due to stronger events causing arousals sooner than weaker events, which in turn may also explain the longer delay from arousal to response after stronger respiratory events. Moreover, the method used in the present study does not detect the peak of the heart rate, but approximately the middle point of the rising slope the timing of which may be affected by different factors. It is not surprising that in a minority of the cases in the present study the PPG response delay was negative, since the heart rate increase may occur even before the arousal [27].

### Clinical Significance

As can be seen in Figures 5 and 6, the variation of the PPG arousal responses within arousal types is large, which may originate for example from the variation between individuals. If that is the case, this variation should be investigated as it might have diagnostic value. Similarly, the significance of intra-individual variation should be studied. Also, the PPG recovery from the arousal responses may hold diagnostic information if, e.g., a less healthy patient is slower to recover. This is valuable as there is a need for novel diagnostic tools for OSA, as the currently used apnea-hypopnea index (AHI) is poorly correlated with the clinical outcomes of OSA [28].

One of the issues with AHI is that it gives the same weight to both apneas and hypopneas, both with and without desaturations [28]. The present study, evaluating the acute cardiovascular effects of arousal-causing respiratory events, may give perspective on how the different types of respiratory events should be emphasized in the diagnosis of OSA.

Ongoing efforts exist to use PPG data for automated scoring of sleep, as using PPG instead of EEG would allow for simpler sleep study setups. For example, automated sleep staging can reliably be conducted based on the PPG signal [29]. It has also been shown that the PPG activity can be used for arousal detection [26], [30] and the present study supports this idea. This may solve a problem with self-applied home OSA tests, where the lack of EEG-based arousal detection leads to fewer hypopneas being scored, causing a different AHI than would be achieved in PSG [28]. Azarbarzin et al. [18] showed that the cardiovascular response also contains information on the arousal intensity. As obstruction type and the presence of desaturation, found to intensify the arousal response in the present study, have also been shown to strengthen the EEG changes in arousal [12], [13], the present study indirectly supports the findings of Azarbarzin et al. [18] with a much larger subject population. The delays between the EEG arousal and the cardiovascular responses should also be taken into account when detecting arousals based on PPG if the timing of the arousals is considered important.

However, there are potential issues with arousal detection based on PPG. Especially in deeper sleep stages, the respiratory events terminate relatively often without a detectable arousal [31]. Even in these cases the vasoconstriction and heart rate increase are present, albeit not as strongly as when an arousal occurs [32]. Even though it has been theorized that this is due to an arousal occurring on a subcortical level, calling these responses arousals has been criticized [19]. Considering that PPG frequency and amplitude changes are sensitive to different aspects that affect arousal intensity, it is possible that combining these PPG characteristics, and possibly others, together might help differentiating other autonomous responses from true arousals. Machine learning may be used to find the optimal combination of characteristics.

### Limitations

This study has certain limitations. First, arousals caused by central or mixed apneas or limb movements were excluded. Second, the chosen metrics for PPG frequency and amplitude were different from those typically used in the literature, limiting their comparability. Moreover, the one-second window used in the derivation of the frequency metric ensured good time resolution, but decreased the frequency resolution compared to using a wider window. However, this is somewhat alleviated by the averaging of the frequency. It is also assumed that the higher harmonic frequencies of the heart rate contribute to the metric. Another limitation is that the PPG segments were not checked for artefacts caused for example by movement. However, it is assumed that the fitting of multiple logistic functions to the signals should in most cases separate artefacts from the actual arousal responses. On the other hand, the 5% considerability limit for the fitted changes was chosen arbitrarily and the sensitivity of the results to the value was not tested, which is a limitation in itself. Additionally, the intra- and interobserver reliability of arousal scoring has been found limited [33], [34]. As the exact timing and duration of the arousals are not diagnostically significant, this unreliability may extend to the timing of the scored arousals, limiting the quality of the arousal response delay analysis. Also, in the subject selection of this study, the comorbidities and medications of the subjects were not considered. This is especially noteworthy in the case of vasoactive medication, which may affect the vasoconstriction responses studied. The dataset does not contain complete and consistent information on the subjects’ medications, so this limitation cannot be overcome and warrants further studies. Finally, a small number of unsuccessful arousal fits were discarded. This is not considered to be a significant limitation due to the small amount of these cases.

## V. Conclusion

The arousals from sleep often elicit heart rate increase and vasoconstriction. The magnitude of the heart rate increase was found to be larger following respiratory arousals than spontaneous ones, and among the respiratory arousals larger following those caused by apneas than those caused by hypopneas. The magnitude of the vasoconstriction was found to be larger if the arousal-causing respiratory event was associated with blood oxygen desaturation. The delay of these cardiovascular responses relative to the arousal was found to be longer following arousals caused by stronger respiratory events. These responses could be used for detecting arousals from the PPG signal, which is easier to measure than EEG, especially in home setting. The individual variation of the response strengths and delays could also hold diagnostic information on the OSA severity.

## Supplementary Materials

Supplementary materials
