# Supplementary material for: Variation in the Photoplethysmogram Response to Arousal From Sleep Depending on the Cause of Arousal and the Presence of Desaturation
Source: IEEE J Transl Eng Health Med. 2024 Jan 4;12:328–39. doi: 10.1109/JTEHM.2024.3349916 (PMC10914203; doi:10.1109/JTEHM.2024.3349916)
Supplement: Supplementary materials [file supp1-3349916.pdf]

Variation in the photoplethysmogram response to arousal from sleep depending on the cause of arousal and the presence of desaturation: Supplementary material

Mieli Luukinen[1,2], Henna Pitkänen[1,2], Timo Leppänen[1,2,3], Juha Töyräs[1,3,4], Anna Sigridur Islind[5], Samu Kainulainen[1,2], Henri Korkalainen[1,2]

[1] Department of Technical Physics, University of Eastern Finland, Kuopio, Finland

[2] Diagnostic Imaging Center, Kuopio University Hospital, Kuopio, Finland

[3] School of Information Technology and Electrical Engineering, The University of Queensland, Brisbane, Australia

[4] Science Service Center, Kuopio University Hospital, Kuopio, Finland

[5] Department of Computer Science, Reykjavik University, Reykjavik, Iceland

Algorithmic description

This section gives a more detailed description of some of the methods of the study. To understand them in their context, the main article, including its methods section, should be read first.

First, information about the arousals and other events was compiled from the sleep scorings for each patient. For each event the following details were listed: starting time, duration, number of 30 s epoch, blood oxygen saturation and desaturation, and sleep stage. Different kinds of arousals and respiratory events were listed. The respiratory and spontaneous arousals were initially accepted to be analyzed if their durations were between 3 s and 15 s, exclusive, and if their endpoints were within the scored epochs.

Then, related respiratory events were assigned to the respiratory arousals to be analyzed. For each arousal, a hypopnea or an obstructive apnea could be considered related to the arousal if it ended later than 5 s before the arousal start but no later than the arousal end. Arousals were discarded if they had zero or more than one related respiratory events.

If an arousal had no sleep stage scoring or happened during an epoch scored as wake, it was assigned the stage of the previous 30 s epoch. If even after that the arousal had no assigned sleep stage, the arousal was discarded. Arousals were also discarded if the related event had no properly defined blood oxygen saturation or had a duration of over 10 s.

For each arousal it was checked that 10 s before and after the arousal was undisturbed by other arousals or respiratory events other than the related event of the arousal. This was done by going through all arousals (not only those selected for analysis) and all respiratory events, including hypopneas and obstructive, central and mixed apneas, and checking whether their start or ending point were within 10 seconds of the arousal. If disturbaces were found around an arousal, that arousal was discarded. The numbers of arousals and patients discarded for each reason are listed in the main article.

When the arousals to be analyzed were chosen and the related events of the respiratory arousals listed, the arousals were divided into types. Spontaneous arousals formed one type and the respiratory arousals were further divided into four types (Table S.I).

TABLE S.I
THE AROUSAL TYPES FOR STATISTICAL ANALYSES

Type	Description
Apnea, desaturation	Respiratory arousal, the related event is apnea that is associated with ≥3% blood oxygen desaturation.
Apnea, no desaturation	Respiratory arousal, the related event is apnea that either is not associated with blood oxygen desaturation or is associated with <3% desaturation
Hypopnea, desaturation	Respiratory arousal, the related event is hypopnea that is associated with ≥3% blood oxygen desaturation.
Hypopnea, no desaturation	Respiratory arousal, the related event is hypopnea that either is not associated with blood oxygen desaturation or is associated with <3% desaturation
Spontaneous	Scored as spontaneous arousal

The respiratory event is related to arousal if the event ends no earlier than 5 s before the EEG arousal start and no later than the EEG arousal end. Arousals were discarded if they had zero or more than one related respiratory events.

The two signal characteristics, mean instantaneous frequency and instantaneous amplitude, were calculated around each arousal and fitted to the function of Eq. 2 in the main text as follows. The slice of the photoplethysmogram signal starting 10 s before the electroencephalogram arousal start and ending 10 after the electroencephalogram arousal end, with a sample rate of 64 Hz, was separated. If this slice would extend outside the measured signal, the fit values were marked as Not a Number (NaN). Spectrogram of the signal slice was calculated using the function `scipy.signal.spectrogram`, with 128 samples per segment, segment overlap of 127 samples, zero-padded segment length of 1024 samples and a boxcar window. The mean instantaneous frequency was then calculated following Eq. 1 of the main text and then smoothing it with a 0th order Savitzky-Golay filter (*i.e.* a moving average filter) with a two-second window.

An initial fitting of Eq. 2 (in the main text) was done using the function `scipy.optimize.curve_fit`, with initial guesses of a, b_1, $b_{2,rel}$, and $b_{3,rel}$ as in Table II (in the main text) and c_1 = -5 s, $c_{2,rel}$ = 0.5 and $c_{3,rel}$ = 0.9 and the fitted parameters are stored. If the fit failed, all the fit parameters were stored as NaN. Then the fitting was repeated with each initial guess combination of Table II, and if the mean square error of the fit was smaller than that of the stored fit, or the stored fit contained NaN values, the stored parameters were replaced with the new fit.

The instantaneous amplitude was defined as the difference of the functions `scipy.ndimage.maximum_filter` and `scipy.ndimage.minimum_filter`, both with a 2 s window, and also then filtered with the 2 s moving average filter. Eq. 2 was then fitted to the amplitude following the same protocol as with the frequency.

The fits of the PPG frequency and amplitude for each arousal were classified, and the response delays, values before and after responses and the presences of recoveries were interpreted based on the classification as described in Table S.II. Fig. S.1. shows examples of the classifications. The magnitude of a response was defined as the difference between the values before and after the response, as defined in the table.

To collect the stable sleep samples, a list of all sleep stability interruptions was constructed for each patient. This list contained all the scored events, with their start and end points, and the sleep stage changes marked at 30 s epoch boundaries with equal start and end points. A time pointer was initialized at 30 s, *i.e.* at the end of the first epoch. The list of interruptions was then traversed through in chronological order. If an interruption started later than the current position of the time pointer and ended within the polysomnogram epochs, the following steps were taken. As many non-overlapping 10 s periods as possible from between the time pointer and the interruption start were saved as stable sleep samples, with the remainder at the end discarded. Then the time pointer was moved to the end of the interruption. Similarly, if the interruption end was later than the current position of the time pointer, the time pointer was moved to the end of the interruption.

Only a subset of the stable sleep samples collected was used in the analyses. To choose this subset, each sample was associated with its sleep stage, as were the respiratory arousals. For each patient and each sleep stage (of REM, N1, N2 and N3) either all the stable sleep samples or a random selection of them corresponding to the amount of that patient's respiratory arousals in that sleep stage were taken, whichever was the smaller amount. For these chosen samples, the mean instantaneous frequency and the instantaneous amplitude were calculated similarly as for the arousal samples, and

the mean value for the sample was taken of both characteristics for the analyses.

TABLE S.II
CLASSIFICATION AND INTERPRETATION OF THE PPG FITS

First step direction	Step considerable: First	Second	Third	Cat. num.	Response delay	Value before response	Value after response	Recovery
Expected	Yes	Yes	Yes	1	If the response area* of the first step is higher than that of the third step (case 1): t_1<br>Otherwise (case 2): t_3	$f(t < t_1)$	Case 1: $f(t_1 < t < t_2)$<br>Case 2: $f(t > t_3)$	Case 1: Yes<br>Case 2: No
			No	2	t_1	$f(t < t_1)$	$f(t_1 < t < t_2)$	Yes
		No	Yes	3	Weighted average of t_1 and t_3 **	Mean before the weighted average of t_1 and t_3 **	Mean after the weighted average of t_1 and t_3 **	No
			No	4	t_1	$f(t < t_1)$	mean($f(t > t_1)$)	No
	No	Yes	Yes	5	If the third step crosses the mean fit value before the second step prominently***: t_3 (response)<br>Otherwise: Undef. (no response)	If response: mean($f(t < t_2)$)<br>If no response: mean($f(t)$)	If response: $f(t > t_3)$<br>If no response: mean($f(t)$)	If response: No<br>If no response: Undef.
			No	6	Undef. (no response)	mean($f(t)$)	mean($f(t)$)	Undef.
		No	Yes	7	t_3	mean($f(t < t_3)$)	$f(t > t_3)$	No
			No	8	Undef. (no response)	mean($f(t)$)	mean($f(t)$)	Undef.
Unexpected	Yes	Yes	Yes	9	If the second step crosses the value in the fit beginning prominently***: t_2 (response)<br>Otherwise: Undef. (no response)	If response: $f(t < t_1)$<br>If no response: mean($f(t)$)	If response: $f(t_2 < t < t_3)$<br>If no response: mean($f(t)$)	If response: Yes<br>If no response: Undef.
			No	10	If the mean after the second step surpasses the value in the fit beginning prominently***: t_2 (response)<br>Otherwise: Undef. (no response)	If response: $f(t < t_1)$<br>If no response: mean($f(t)$)	If response: mean($f(t > t_2)$)<br>If no response: mean($f(t)$)	If response: No<br>If no response: Undef.
		No	Yes	11	Undef. (no response)	mean($f(t)$)	mean($f(t)$)	Undef.
			No	12	Undef. (no response)	mean($f(t)$)	mean($f(t)$)	Undef.
	No	Yes	Yes	13	t_2	mean($f(t < t_2)$)	$f(t_2 < t < t_3)$	Yes
			No	14	t_2	mean($f(t < t_2)$)	mean($f(t > t_2)$)	No
		No	Yes	15	Undef. (no response)	mean($f(t)$)	mean($f(t)$)	Undef.
			No	16	Undef. (no response)	mean($f(t)$)	mean($f(t)$)	Undef.

For frequency, the expected direction is upwards, and for amplitude downwards. To be classified as considerable, a step must have a magnitude of 5 % of the mean fit value. A step may also be deemed not considerable if non-considerable steps either by sum of their magnitude or due to the time between steps lead to the measured values before and after the response to differ in the unexpected direction.

The response delay is measured relative to the EEG arousal start (unit s). The unit for frequency is Hz and for amplitude arbitrary unit. When a mean of the fit or a part of it is taken, an unrounded version of (1) is used, with Heaviside step functions substituted for the logistic functions.

f(t), the fitted function of time; t_1, t_2, t_3, times of the first, second and the third step, respectively, Undef., undefined.

* The response area of the first step is defined as the time between the first and second steps multiplied by the difference of the value in the fit beginning and the value between the first and second steps. The response area for the third step is the time between the third step and the fit end multiplied by the difference of the value in the fit beginning and the value after the third step.

** If the first and third steps are deemed considerable, but the second is not, the first and third step are interpreted as one change. The time of this change is determined by taking a weighted average of the step times, using step magnitudes as weights, adjusted so that the magnitude of the second step is subtracted from the considerable step closer to it in time.

*** Prominently crossing a value is here defined by crossing it by more than 5% of the mean of the whole fit.

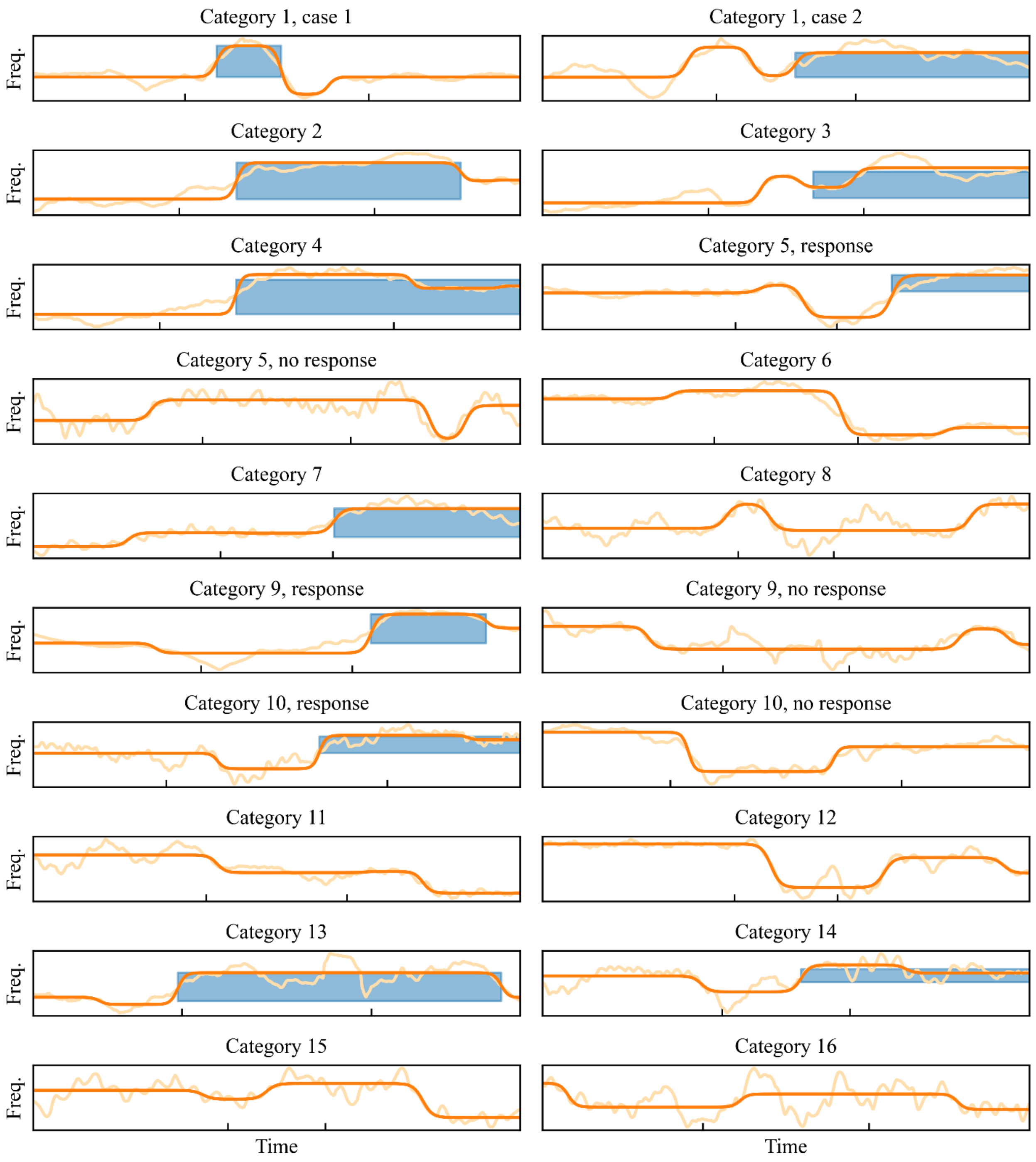

Fig. S.1. Examples of mean instantaneous frequency fits in each category and subcategory listed in Table S.I. The light orange line shows the smoothed mean instantaneous frequency, the darker orange line is the fitted function and the blue rectangle marks the detected response. The ticks on the time axes show the start and end times of the EEG arousals. The time range of each subfigure extends from 8 s before the EEG arousal start to 8 s after the EEG arousal end. Thus the time scales are different in each subfigure. Similarly, the frequency scales also differ.
